# The Ecology of Benthic Diatom Assemblages in Saline Wetlands of the Ebro Basin, NE Spain

**DOI:** 10.1007/s00248-025-02514-3

**Published:** 2025-03-17

**Authors:** S. Blanco, R. Viso, M. Borrego-Ramos, R. López-Flores, D. Mota-Echeandía, M. Tierra, J. Herrero, C.Castañeda

**Affiliations:** 1 Diatom Lab, University of León, La Serna St., 58, 24007 León, Spain; 2 Department of Agricultural and Environmental Natural Sciences, Technological College, University Institute for Research in Environmental Sciences of Aragon (IUCA), University of Zaragoza, Zaragoza, Spain; 3 Estación Experimental de Aula Dei, EEAD-CSIC, Av. Montañana 1005, 50059 Zaragoza, Spain

**Keywords:** Electrical conductivity (EC), Species richness, Ecological niche, Community structure, Ebro Basin, Aridity

## Abstract

Benthic diatoms play a crucial role in aquatic ecosystems as indicators of environmental conditions and contributors to primary productivity. This study explores the ecology of benthic diatom assemblages in saline wetlands in NE Spain, focusing on the relationships between community parameters, species distributions, and environmental factors, particularly conductivity. Samples were collected from several wetlands representing a range of conductivity and trophic state. A total of 25 diatom taxa were identified, with assemblages dominated by halophilous species. Non-metric multidimensional scaling analysis revealed electrical conductivity (EC) as a primary factor shaping diatom communities, with nutrient levels as a secondary influence. Species exhibited varying responses to the EC gradient, with some showing overlapping niches and others clearly separated. The study found strong correlations between species abundance, occupancy, and their contribution to dissimilarity between sampling sites. More abundant and widespread species were key drivers of community structure and differentiation. Additionally, a significant relationship was observed between taxa occurrence and niche breadth, measured as EC tolerance. Species with broader tolerances tended to have higher occupancy rates, supporting ecological theories about generalist strategies in variable environments. Contrary to some previous research, rare taxa (3–5% in relative abundance) had a negligible effect on assemblage segregation among habitats. The findings suggest that both environmental filtering based on EC tolerance and species’ inherent characteristics play important roles in shaping diatom community composition across these saline wetlands. This study contributes to our understanding of diatom ecology in saline habitats and highlights the importance of considering both local abundance and environmental tolerance in ecological studies of these communities. The insights gained can inform more accurate ecological models and improve our understanding of species distribution and community dynamics in saline aquatic ecosystems.

## Introduction

Benthic diatoms are a crucial component of aquatic ecosystems, acting as indicators of environmental conditions and contributing to the primary productivity of these habitats. The ecology of benthic diatoms in saline habitats is primarily driven by electric conductivity (EC) levels, with distinct communities forming in different conductivity regimes. Diatom species exhibit varying conductivity tolerances, with some thriving in hypersaline conditions while others prefer brackish environments. EC drives assemblage composition and, thus, community structural parameters, mainly species richness and dominance [1]. Hence, this variable is considered as an excellent surrogate of ecological status in continental ponds [2]. In southern Spain, Fernández-Moreno et al. [3] categorized inland waters into oligosaline, mesosaline, and eusaline based on their EC levels, with distinct dominant species in each group, but factors such as habitat complexity, nutrient content, and biotic interactions also play a role in shaping the spatial patterns of benthic diatom assemblages inhabiting these systems [4].

Of particular interest within diatom ecology is the study of the realized ecological niche. It has been demonstrated that broader niches correlate with wider distributions and higher local abundances, while more marginal niches lead to restricted distributions [5]. Blanco et al. [6] have recently highlighted the importance of individual species size and dispersion abilities as drivers of diatom community structure in Mediterranean ponds, showing that the positive abundance-occupancy relationship in these communities may reflect structural features beyond the simple pattern of widespread species being abundant and narrowly distributed species being rare [7, 8].

These findings underscore the complex interplay of natural and anthropogenic factors in determining the characteristics of benthic diatom communities in saline ponds. However, while diatoms serve as indicators of ecological health, their assemblage structure may not always correlate directly with overall pond quality, as seen in the contrasting responses of macroinvertebrates [9]. Besides, while the ecological roles of benthic diatoms are well-documented, ongoing environmental changes, such as salinization, may disrupt these communities, necessitating further research to understand their resilience and adaptability. This paper explores the statistical relationship between community-level parameters such as abundance, occurrence, and average dissimilarity along a conductivity gradient in benthic diatoms collected from Spanish inland saline wetlands. Research (e.g., Melo, 2021) indicates that rare species often play a significant role in distance metrics, sometimes more so than abundant species, so we specifically address the contribution of diatom species to dissimilarity between sampling sites.

## Materials and Methods

### Study Sites

**Fig. 1:**
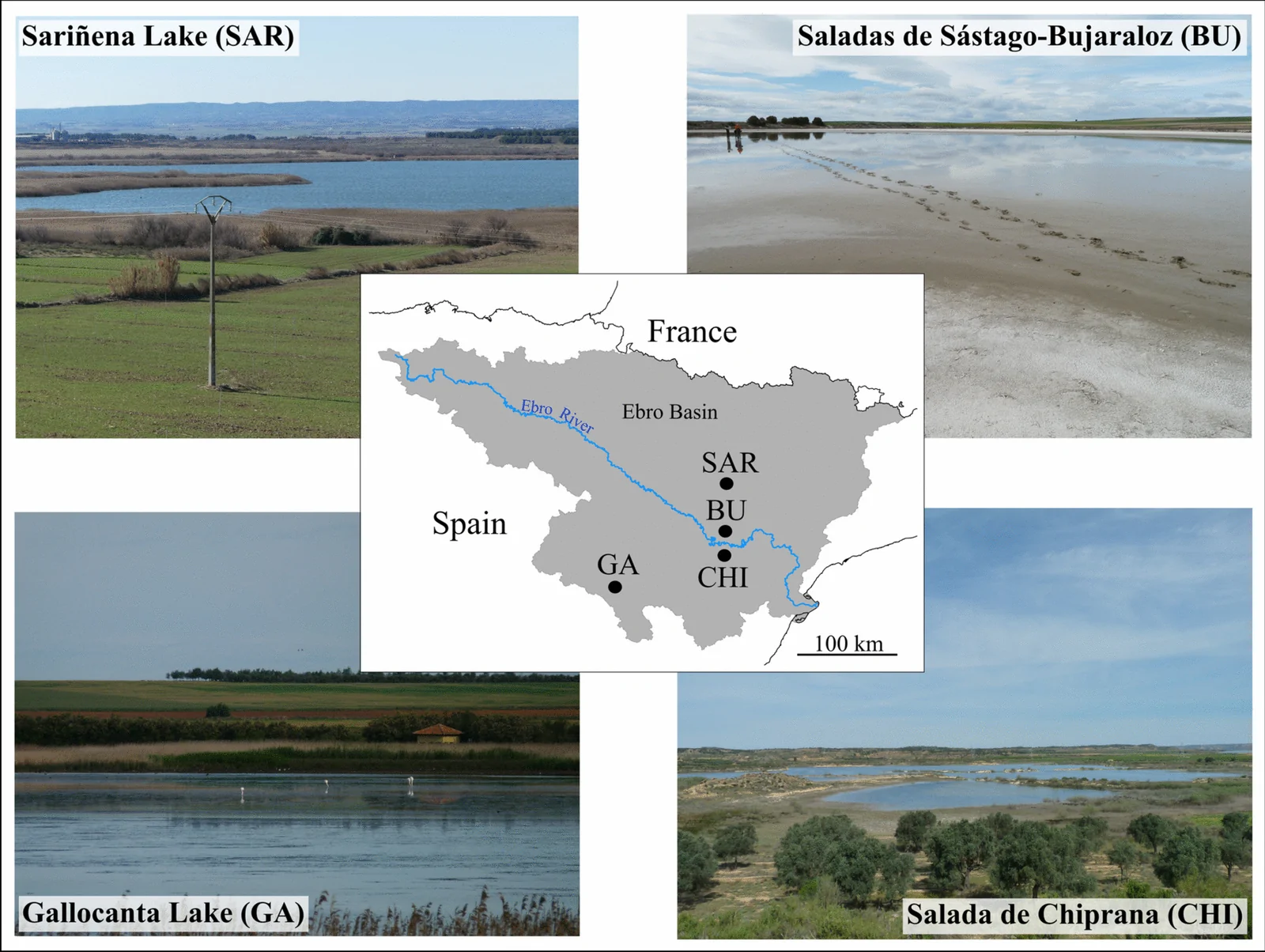
Views of the study sites, and their location in the Ebro Basin, Spain

The saline wetlands studied are located in the Ebro Basin, NE Spain (Fig. 1). The wetlands are Salada de Chiprana (hereafter Chiprana), Gallocanta Lake, Saladas de Sástago-Bujaraloz (hereafter Saladas), and Sariñena Lake. All of them are cataloged in the Natura 2000 European Network and three of them are included in the Ramsar list of wetlands of International importance (Ramsar Convention Secretariat, 2010). Besides, they are protected by the European Water Framework Directive (91/676/EC) as vulnerable waters against pollution. Saladas (including La Playa and Salobral ponds) is a complex of almost 150 close depressions from less than 1 ha to 200 ha, of aeolian and karstic origin, some of them hosting saline wetlands of playa-lake type disappearing due to agricultural intensification [10]. Chiprana, with 31 ha, is the only permanent hypersaline lake in SW Europe. Sariñena is a permanent lake of 130 ha resulting from the degradation of a previous fluctuating saline wetland, with interest for the nesting of aquatic birds.

The wetlands studied represent a gradient regarding the water conductivity, their hydrology, and the agricultural intensification in their watersheds. Chiprana and Sariñena lakes are permanent, whereas Gallocanta and Saladas fluctuate until getting dried conditions, especially evident and longer in the last decades. The free water presence of Saladas playa-lakes varied from 3 to 6 months in a wet year [11]. Gallocanta lake can reach a surface extent of 12 km^2^ and experience periods of complete desiccation (Palomar-Vázquez et al., 2022). Chiprana receives fresh water inputs through ditches and subsurface irrigation return flows. Sariñena has been fed by subsurface return irrigation flows from the transformation to irrigation in the 1950s [12].

The climate in the Ebro Basin (Saladas, Chiprana, Sariñena) is Mediterranean with a mean annual temperature ranging from 14.9 to 25 ºC and 11.2 ºC in Gallocanta. The mean annual rainfall, between 316 and 480 mm, together with the high evapotranspiration (ET_0_), between 1050 and 1500 mm, characterize these semiarid environments.

### Field and Laboratory Procedures

Samples were taken during spring 2023. We selected 18 sampling points for diatoms and the corresponding water characterization, distributed in the four study sites. Diatom samples were taken by placing polypropylene ropes as submersed artificial substrata for benthic diatoms during 6 weeks. Water was sampled at the end of this 6-week period, only in 12 out of the 18 sampling points because some points had dried out.

Field determinations included the water temperature (°T), pH, and electrical conductivity (EC), which were measured using a portable multimeter CRISON MM26+. In the laboratory, the samples for the analysis of dissolved inorganic nutrients were filtered using Whatman GF/F filters and refrigerated at temperatures below 5 ºC until analysis. Total nitrogen (TN) was determined directly from the refrigerated samples. Analytic methods were based on APHA/AWWA/WEF. Main anions were determined by ion chromatography (Metrohm). For the analysis of planktonic chlorophyll-*a* concentration, a known volume of water samples was collected and filtered using a Whatman GF/F filter, then preserved at − 20 ºC. Subsequently, extraction was performed using 90% acetone, and spectrophotometric analysis was conducted following the method of Jeffrey et al. [13].

Sampling method follows the protocol for the collection of periphytic diatoms in shallow lakes described by Blanco and Bécares [14]. Samples were preserved in plastic bottles by adding 5% formaldehyde. In the lab, biofilm was removed from the ropes by gently shaking in distilled water during 1 min [15]. Resulting suspensions were cleaned by oxidation with hot hydrogen peroxide 30% v/v and then rinsed three times by decantation using distilled water. Air-dried aliquots were mounted on permanent glass slides using the refractive resin Naphrax® according to standard European protocols (UNE-EN 13946). On each slide, ca. 100 diatom valves were counted and identified until the lowest possible taxonomic level (species, subspecies, variety or form) with a Leica DMR microscope (Leica, UK) equipped with differential interference contrast (DIC) and an Optika digital camera, based on Álvarez-Blanco & Blanco (2014), Witkowski et al. (2000) and references therein. Ropes were measured in order to obtain relative cell densities (per mm^2^ of substratum). Due to the sample size considered, hereafter “rare” taxa will include species with relative abundances between 3 and 5%.

The abundance (% of total individual counts) and occurrence (% of samples where present) of each taxon were calculated. In order to assess which diatom taxa were most responsible for differences in assemblage composition among the four sampling sites (Chiprana, Gallocanta, Saladas, and Sariñena), a SIMPER analysis [16] was performed using the Bray-Curtis similarity measure. This analysis calculates the average dissimilarity between all pairs of samples across these predefined wetland sites and identifies the percentage contribution of each taxon to that dissimilarity. The resulting dissimilarity scores per taxon were used to explore relationships with species abundance (percentage of total counts) and occupancy (percentage of samples where present), testing the hypothesis that abundant and widespread taxa drive community differentiation. Finally, the response curves of dominant taxa against the EC gradient were determined by the weighted averaging method according to Braak and van Dam [17], where species tolerances are calculated as:

$$T=\sqrt{\frac{\sum_{i=1}^n(x_{i}-O)^{2}}{\frac{(N^{'}-1)\sum_{i=1}^nw_{i}}{N^{'}}}}$$

where *x*_*i*_ is EC value at site *i*, O is the conductivity optimum, *w*_*i*_ is the abundance of the taxon at site *i*, *n* is number of sites and *N*′ is number of sites where the taxon occurs. Statistical computations and graphics were obtained with PAST software v. 4.17 [18].

## Results

**Fig. 2:**
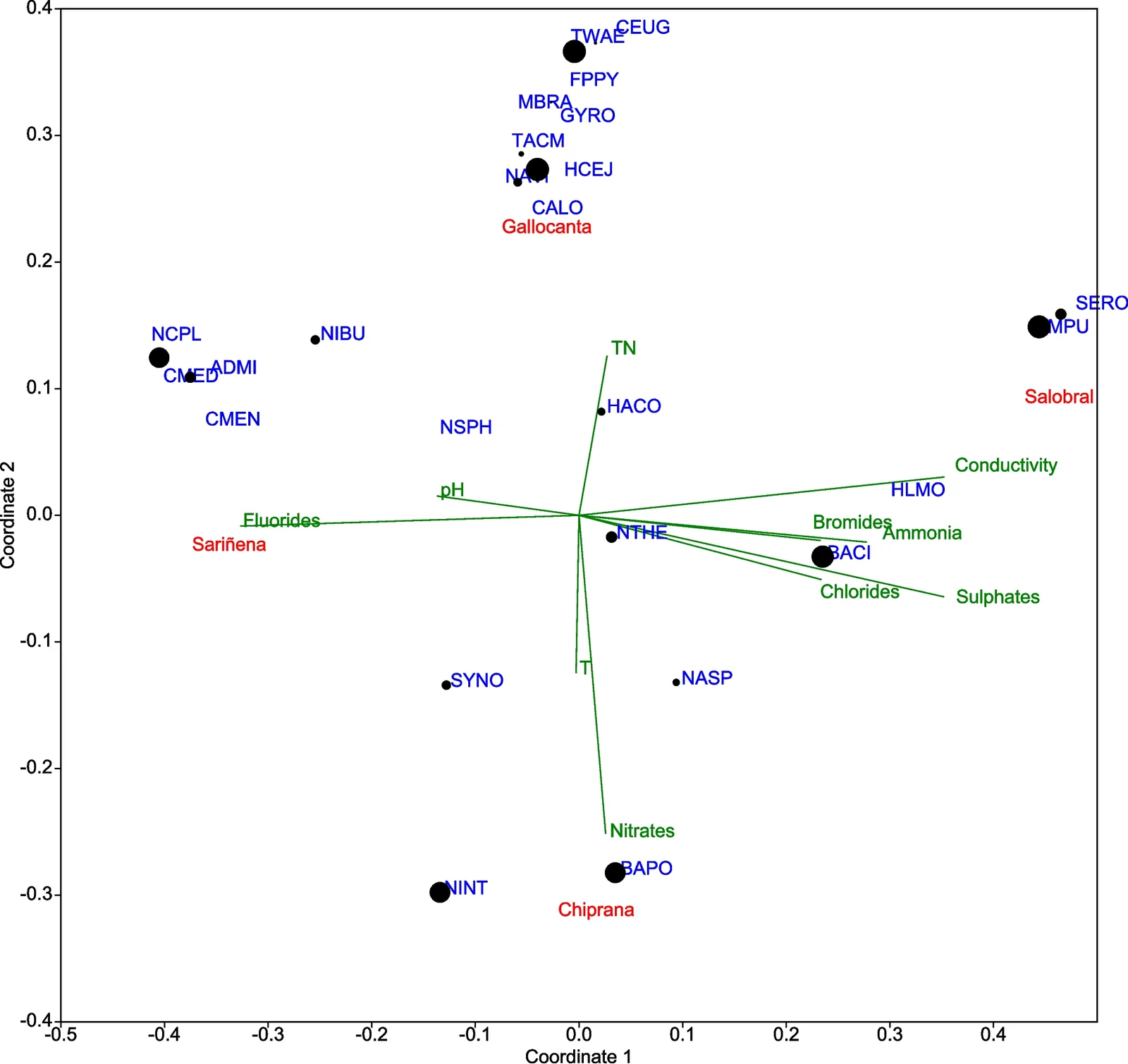
Nonmetric multidimensional scaling triplot showing the relationship between diatom taxa, studied wetlands and environmental variables. Singleton species were excluded. Sampling sites are indicated with red labels, representing sampling point centroids. Circle diameter is proportional to species’ average dissimilarity scores in SIMPER analysis (see text for details). Taxa codes are indicated in Table 3. Stress value = 0.2

With EC values up to ~ 16 dS/m, the analyzed waters could be considered as highly saline, with an electrolyte content mostly dominated by chlorides and sulfates (Table 1). The trophic gradient was also very broad, from oligotrophic waters to hypertrophic systems such as Chiprana (44 ppm of nitrates). A total of 58 different diatom taxa were identified, with assemblages dominated by halophilous species such as *Tabularia waernii*, *Halamphora cejudoae*, or *Seminavis pusilla*, both in terms of absolute abundance and frequency of occurrence. Samples contained on average 10 ± 4 species per 100 individuals, with generally low diversity scores (Shannon’s alpha: 1.31 ± 0.44). Dominance values ranked from 0.43 to 0.72. The distribution of species among sites can be displayed in the NMDS plot (Fig. 2), together with the relative influence of abiotic predictors in the structure of the diatom communities. EC values correlated with most water anions along the first axis, and the corresponding vectors point to the sample collected in Salobral (109.6 dS/m), with large populations of *Seminavis* species. On the contrary, Sariñena samples, with oligohalobous diatom taxa such as *Nitzschia capitellata* or *Cyclotella* sp., were conditioned by fluorides. The secondary axis denotes a nutrient level gradient that clearly separated Gallocanta (mesohalobious) and Chiprana (oligohalobious) diatom assemblages, dominated respectively by *Tabularia waernii* and *Brachysira aponina*.

**Table 1:** Main characteristics of the wetlands studied

	Chiprana (*n* = 3^a^)	Gallocanta (*n* = 4^a^)	Saladas (*n* = 3^b^)	Sariñena (*n* = 2)
Mean annual rainfall, mm	316	480	330	460
Mean annual ET_0_, mm	1300–1500	1050	1300	1200
Elevation, m a.s.l.	137	990	320–330	330
Flooding regime	Permanent	Fluctuating until dry	Fluctuating until dry	Permanent
Depth of water, m	<4.5	<2.8	<0.5	<3

^a^+ 1 dry sample

^b^+ 5 dry samples

**Fig. 3:**
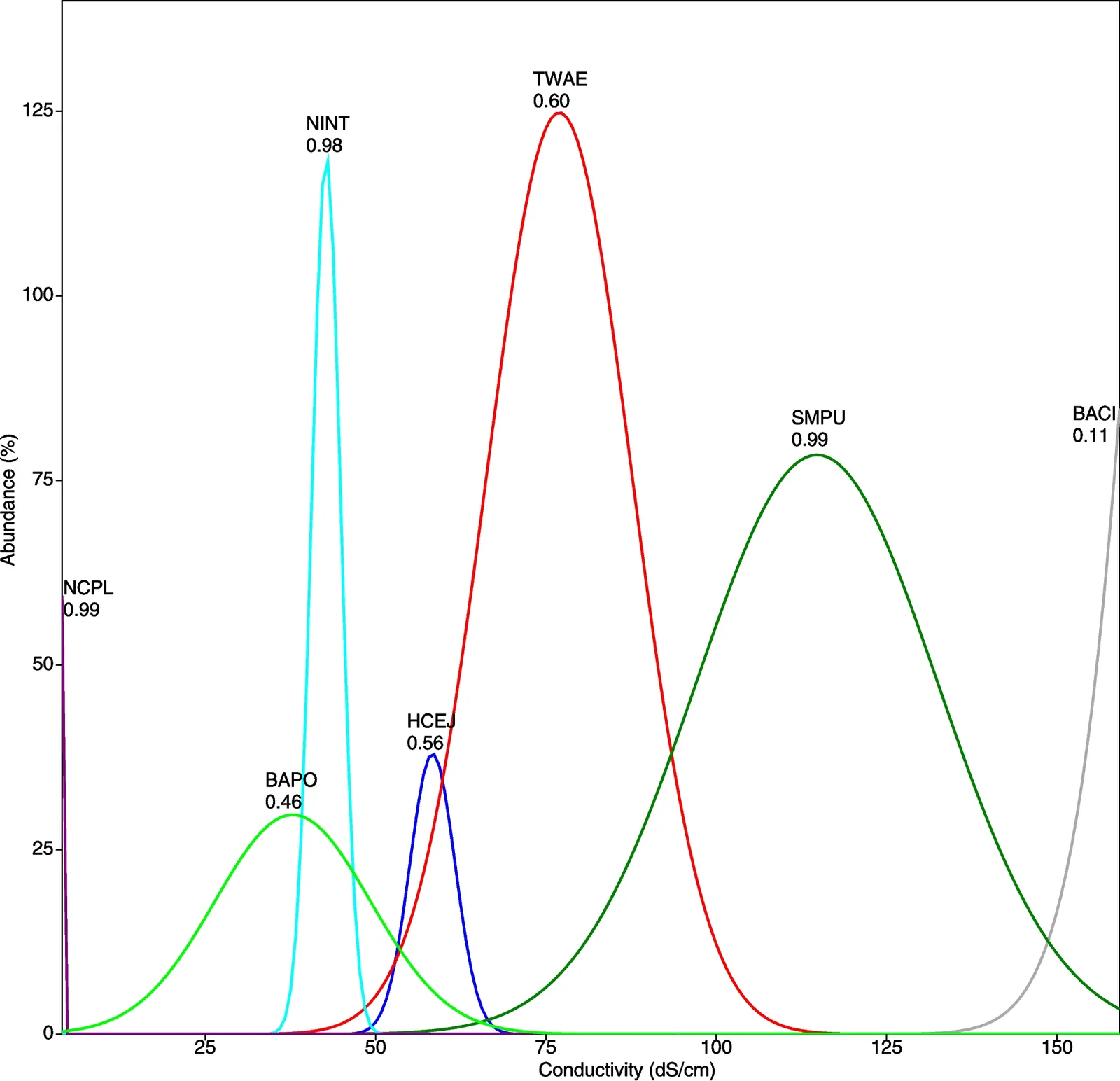
Response curves of main (> 5% in total abundance) species to conductivity levels in the studied systems. Singleton species were excluded. Taxa codes are indicated in Table 3. Numbers indicate gaussian fit *R*^2^ values

Diatom ecological profiles along the conductivity gradient are shown in Fig. 3. Species coexisting in the same habitat tended to exhibit overlapping response curves (e.g., *Halamphora cejudoae* and *Tabularia waernii* in Gallocanta), whereas taxa with very different electrical conductivity optima (e.g., the oligohalobous *Nitzschia capitellata* and the mesohalobous *Bacillaria* sp. tended to occur in habitats clearly separated along the conductivity gradient (Sariñena and La Playa, respectively).

**Fig. 4:**
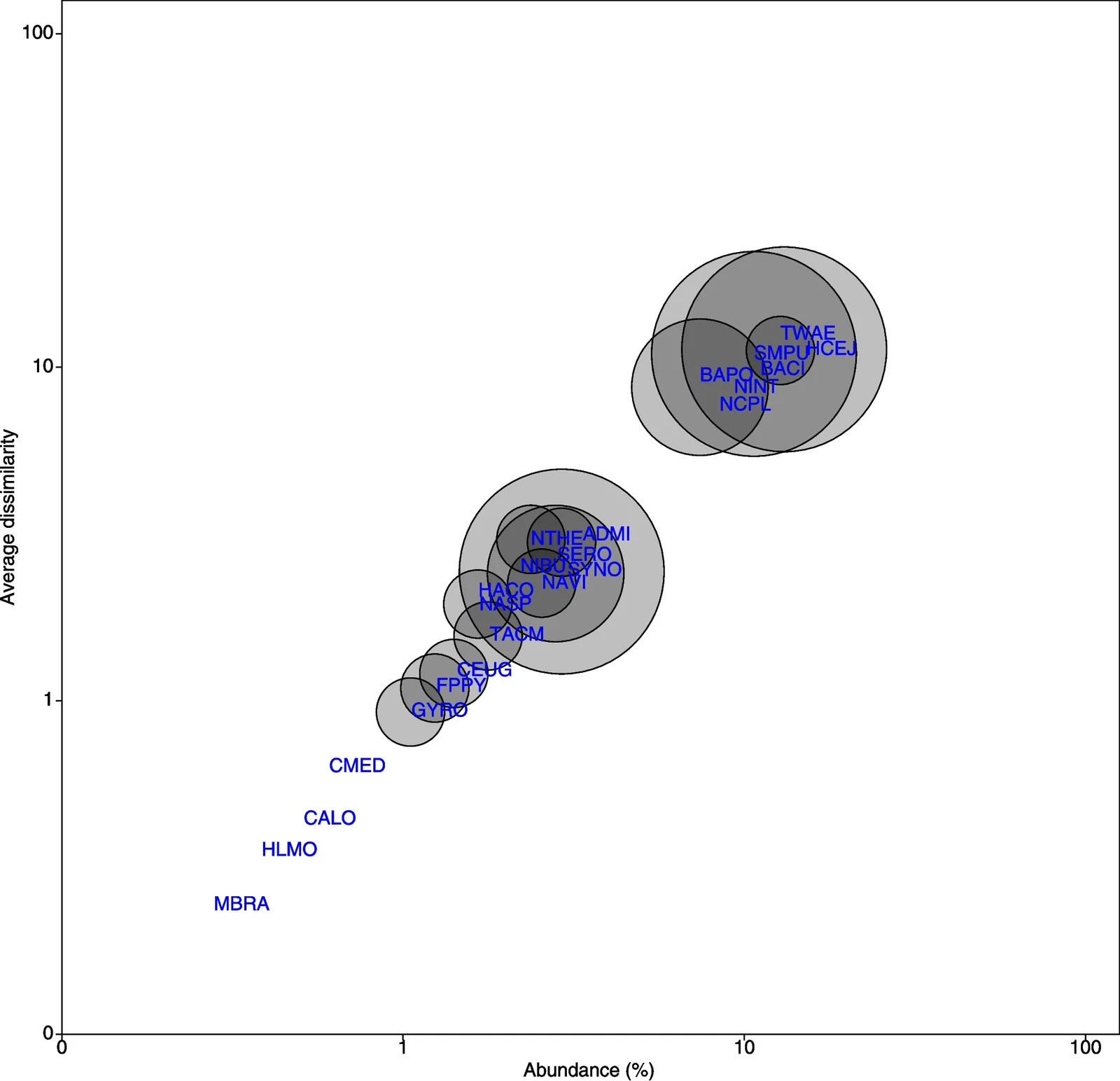
Relationship between taxa abundance, occupancy (expressed as circle diameters), and average dissimilarity. Singleton species were excluded. Taxa codes are indicated in Table 3. Note the log scale used in axes

**Fig. 5:**
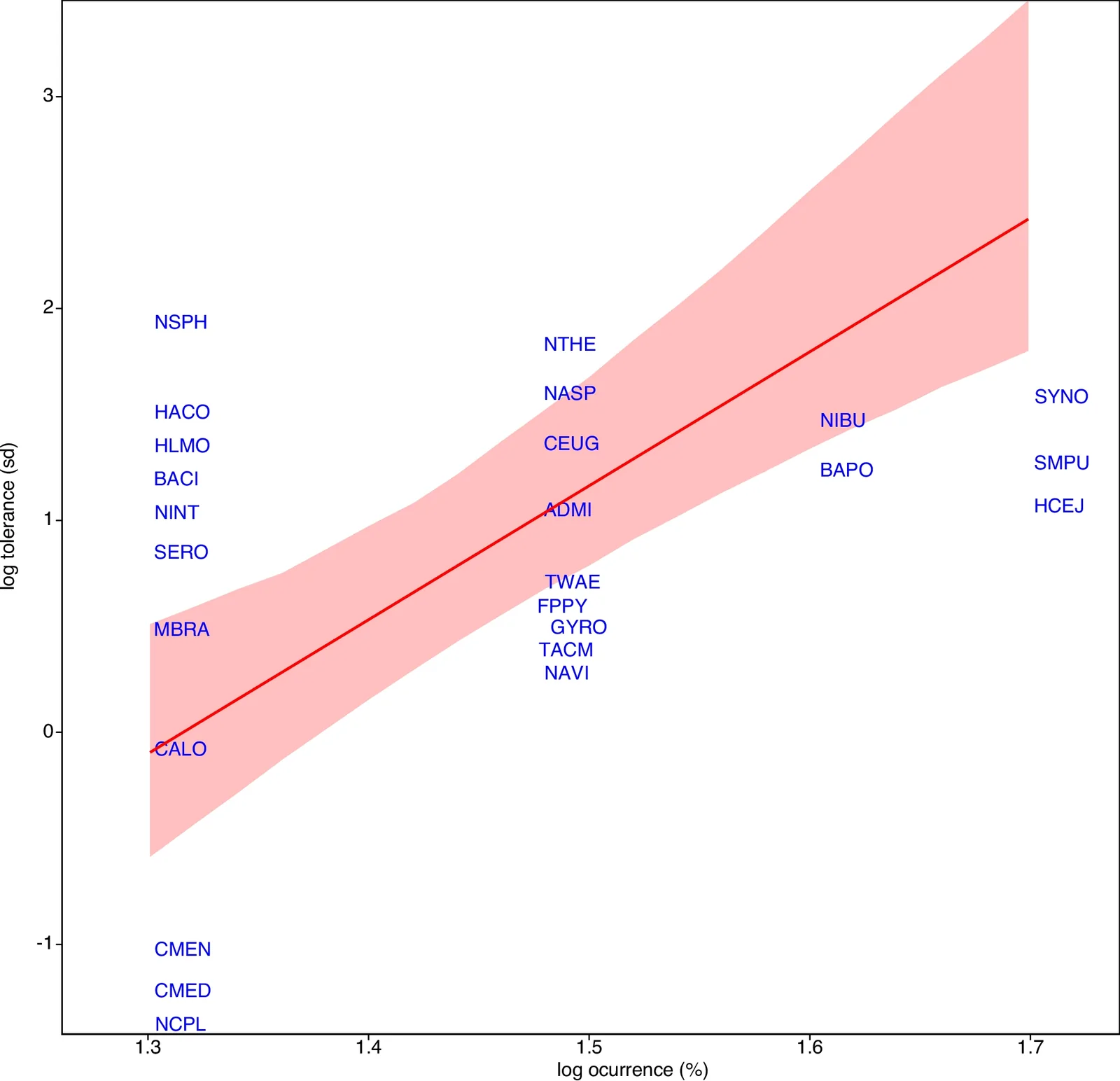
Relationship between taxa occupancy and tolerance to conductivity, measured as standard deviation. Data fitted to a reduced major axis regression line ± 95% confidence limits. Singleton species were excluded. Taxa codes are indicated in Table 3

Figure 4 shows that abundant and widespread taxa strongly influenced site differences in SIMPER analysis (abundance: *R*^2^ = 0.98, *p* < 0.01; occupancy: *R*^2^ = 0.15, *p* = 0.05). SIMPER analysis revealed that species with higher abundance and occupancy, such as *Halamphora cejudoae* and *Seminavis pusilla*, contributed most to the average dissimilarity among the four wetland sites (Fig. 4), supporting their role as key drivers of community structure. There is also a weaker but significant (*R*^2^ = 0.15, *p* = 0.05) relationship between taxa occurrence and niche breadth measured as EC tolerance (weighted standard deviation), as shown in Fig. 5, where species occurring in 50% of samples (*Synedropsis* sp., *Seminavis pusilla*, *Halamphora cejudoae*) exhibit broad response curves against this variable.

## Discussion

The observed range of conductivity and trophic states in these saline wetlands highlights the complexity and diversity of inland aquatic ecosystems occurring in the semiarid NE Spain. In the studied systems, the dominance of chlorides and sulfates indicate that these wetlands are influenced by the composition of the geological materials, the groundwater chemistry, and the hydrological regime [19, 20]. Regarding the diatom community, the identification of 25 diatom taxa indicates moderate species richness in the sampled environment. Diatom species richness in hypersaline wetlands varies considerably, with studies reporting different numbers across various scales. In a single hypersaline lake, 51 species were identified over 14 years [21], while a broader study of salt lakes found 159 taxa in one wetland [22]. In our case, the dominance of halophilous species like *Tabularia waernii*, *Halamphora cejudoae*, and *Seminavis pusilla* suggests saline or brackish habitats. These species are well-adapted to high conductivity conditions, which is consistent with their prevalence in both abundance and occurrence frequency [23]. Their dominance in species assemblages is often observed in stressful or specialized environments, where only a few well-adapted species can thrive. Besides, the wide range of dominance values could reflect spatial or temporal heterogeneity in environmental conditions within the study area (Table 2).

**Table 2:** Descriptive statistics of main environmental features in the studied wetlands

	Median	Min	Max
Electrical conductivity (dS/m)	54.90	3.99	159.20
pH	8.50	7.55	9.07
*T* (°C)	15.60	10.90	22.60
Fluorides (ppm)	0.01	0.01	0.21
Chlorides (ppm)	23,163.50	945.62	126,797.00
Bromides (ppm)	68.70	3.70	201.60
Nitrates (ppm)	1.50	0.35	44.13
Total nitrogen (ppm)	0.03	1.42	3.26
Ammonia (ppm)	0.04	2.24	14.42
Phosphates (ppm)	< 0.02	0.22	18.97
Sulphates (ppm)	12,242.50	355.65	90,312.00
Chlorophyll-*a* (mg/m^3^)	0.22	19.38	308.36

The strong influence of EC indicates that conductivity is a primary factor shaping diatom communities in these sites. Taxa like *Seminavis* are well-adapted to hypersaline conditions, while *Nitzschia capitellata* and *Cyclotella* prefer fresher waters (Table 3). This agrees with previous studies describing the autecological preferences of these diatoms [24, 25]. The separation of Gallocanta and Chiprana lakes along the nutrient axis suggests also that *Tabularia waernii* and *Brachysira aponina* have different nutrient requirements. On the other hand, the clear separation between oligohalobous and mesohalobous species along the EC gradient demonstrates niche differentiation based on conductivity tolerance. This differentiation allows diverse diatom communities to exist across a range of saline environments.

**Table 3:** Species codes used in the figures. Singleton species were excluded

ADMI	*Achnanthidium minutissimum* (Kützing) Czarnecki
BACI	*Bacillaria* sp.
BAPO	*Brachysira aponina* Kützing
CALO	*Caloneis* sp.
CEUG	*Cocconeis euglypta* Ehrenberg
CMED	*Cyclotella meduanae* Germain
CMEN	*Cyclotella meneghiniana* Kützing
FPPY	*Fallacia pygmaea* (Kützing) A.J. Stickle & D.G. Mann
GYRO	*Gyrosigma* sp.
HACO	*Halamphora coffeaeformis* (Agardh) Levkov
HCEJ	*Halamphora cejudoae* Alvarez-Blanco & S.Blanco
HLMO	*Halamphora montana* (Krasske) Levkov
MBRA	*Mastogloia b*r*aunii* Grunow var. braunii
NASP	*Navicula* sp.
NCPL	*Nitzschia capitellata* Hustedt in A. Schmidt et al.
NIBU	*Nitzschia bulnheimiana* (Rabenhorst) H.L.Smith
NINT	*Nitzschia intermedia* Hantzsch ex Cleve et Grunow
NSPH	*Nitzschia spathulata* Brébisson in Wm. Smith
NTHE	*Nitzschia thermaloides* Hustedt
SERO	*Seminavis robusta* Danielidis & D.G. Mann
SMPU	*Seminavis pusilla* (Grunow) E.J. Cox & G.Reid
SYNO	*Synedropsis* sp.
TACM	*Tryblionella acuminata* W.M.Smith var. acuminata
TWAE	*Tabularia waernii* Snoeijs

Our results show that the most abundant and widespread species are key drivers of community structure and differentiation across the studied wetlands. Taxa with higher population sizes and distribution areas contribute significantly to the overall dissimilarity between sampling sites, which aligns with the general understanding that species occupying more sites not only tend to be more locally abundant but also contribute more to dissimilarity across sampling sites [26]. Additionally, the relationship between taxa occurrence and niche breadth, particularly EC tolerance, suggests that species like *Synedropsis* sp. and *Seminavis pusilla* exhibit broad response curves, highlighting their adaptability in varying environmental conditions [27]. These species have a large influence on ecosystem functioning and could be considered keystone species in these environments. Species with wider EC tolerances are able to colonize and persist in a broader range of habitats, leading to higher occupancy rates [28]. These widely occurring species with broad tolerances represent successful generalist strategies in these variable saline environments, and such a relationship between niche breadth and occupancy supports classic ecological theories [29, 30] about the advantages of generalist strategies in variable environments.

Melo [31] argues that properly accounting for and weighting rare species in dissimilarity measures can provide a more accurate picture of community differences, especially for detecting subtle variations between sites or along weak environmental gradients. On the contrary, our findings evidence that rare taxa had a negligible effect on assemblage segregation among habitats. Rare taxa tend to be more sensitive to environmental changes, leading to weaker associations with specific habitat variables compared to common species [32, 33]. Moreover, these species show higher temporal variations and are more influenced by stochastic processes, suggesting that their presence is more transient and less impactful on long-term dissimilarity between sites [34].

The results presented in this paper suggest that both environmental filtering (based on EC tolerance) and species’ inherent characteristics (abundance and occupancy) play important roles in shaping diatom community composition across these arid wetlands. In summary, our findings on species abundance, occupancy, and their contribution to dissimilarity are well-supported by existing literature. Studies consistently found positive correlations between local abundance and regional occupancy [35, 36], but niche position emerges as a strong predictor of species distribution patterns, with more marginal positions associated with restricted spatial distribution and lower local abundance [5, 36]. Niche breadth also influences occupancy and abundance, with broader niches linked to wider distribution and greater local abundance [5]. In our case, the strong predictors of dissimilarity and the relationship between taxa occurrence and niche breadth highlight the importance of considering both local abundance and environmental tolerance in ecological studies. These insights can inform more accurate ecological models and improve our understanding of species distribution and community dynamics. 

## Data Availability

No datasets were generated or analysed during the current study.
